# Interleukin-6, CXCL10 and Infiltrating Macrophages in COVID-19-Related Cytokine Storm: Not One for All But All for One!

**DOI:** 10.3389/fimmu.2021.668507

**Published:** 2021-04-26

**Authors:** Francesca Coperchini, Luca Chiovato, Mario Rotondi

**Affiliations:** ^1^ Laboratory for Endocrine Disruptors, Unit of Internal Medicine and Endocrinology, Istituti Clinici Scientifici Maugeri IRCCS, Pavia, Italy; ^2^ Department of Internal Medicine and Therapeutics, University of Pavia, Pavia, Italy

**Keywords:** COVID-19, macrophages, IL-6, CXCL10, cytokine-storm

## Abstract

SARS-COV-2 virus is responsible for the ongoing devastating pandemic. Since the early phase of the pandemic, the “cytokine-storm” appeared a peculiar aspect of SARS-COV-2 infection which, at least in the severe cases, is responsible for respiratory treat damage and subsequent multi-organ failure. The efforts made in the last few months elucidated that the cytokine-storm results from a complex network involving cytokines/chemokines/infiltrating-immune-cells which orchestrate the aberrant immune response in COVID-19. Clinical and experimental studies aimed at depicting a potential “immune signature” of SARS-COV-2, identified three main “actors,” namely the cytokine IL-6, the chemokine CXCL10 and the infiltrating immune cell type macrophages. Although other cytokines, chemokines and infiltrating immune cells are deeply involved and their role should not be neglected, based on currently available data, IL-6, CXCL10, and infiltrating macrophages could be considered prototype factors representing each component of the immune system. It rapidly became clear that a strong and continuous interplay among the three components of the immune response is mandatory in order to produce a severe clinical course of the disease. Indeed, while IL-6, CXCL10 and macrophages alone would not be able to fully drive the onset and maintenance of the cytokine-storm, the establishment of a IL-6/CXCL10/macrophages axis is crucial in driving the sequence of events characterizing this condition. The present review is specifically aimed at overviewing current evidences provided by both *in vitro* and *in vivo* studies addressing the issue of the interplay among IL-6, CXCL10 and macrophages in the onset and progression of cytokine storm. SARS-COV-2 infection and the “cytokine storm.”

## Introduction

The devastating epidemic caused by SARS-COV-2 prompted the scientific community to investigate the behavior of SARS-COV-2 by both *in vitro* and *in vivo* studies. In these last months we have progressively accumulated more and more information regarding SARS-COV-2 biological behavior. In particular, it is now known, that it is a RNA virus which interacts with the ACE-2 receptor expressed in several human tissues (1–7). This binding is followed by a cleavage of the S spike protein of the virus by the two proteases Furin and TMRPRLS leading to the entering of SARS-COV2 through the membrane of the host cell, in which it replicates. The virus not only infects the upper respiratory tract but the infection also involves other tissues like gastrointestinal tract and endothelial cells. The host immune response to the virus is, at least in the most severe cases, ineffective due to the ability of the virus to by-pass the interferon-mediated anti-viral immunity, being associated with a reduction of T cells to lymphopenia and to an immune-deviation to a Th17 phenotype that is inappropriate for successful virus defence. COVID-19 clinical course ranges from asymptomatic cases to very severe disease and septic status which may ultimately lead to multi organ failure (8, 9). A main focus was made in these months to a peculiar characteristic of SARS-COV-2 infection, which is the induction of the so-called cytokine storm (CS). CS is a clinical situation in which the immune system releases (in the case of SARS-COV-2 mainly the lung) a wide spectrum of immune-active molecules, including cytokines and chemokines at very high concentrations, potentially driving the onset of multi-organ failure and in the most unfortunate cases death.

Based on our current knowledge, the balance of the composition of the cytokine/chemokine network characterizing the “cytokine storm” seems to be crucial in the progression of COVID-19 (10, 11).

Following the demonstration of the pathological role of the cytokine storm, several studies were designed with the aim of investigating whether specific chemokines could play a role in driving COVID-19 progression. These type of studies were aimed not only at identifying possible targets for treatment of the disease but also at detecting a possible “immune signature” in patients with COVID-19 (11). Indeed, COVID-19 is a very heterogeneous disease, characterized by a clinical course which may range from a rather free of symptoms condition to fatal events. Thus, it is evident that the identification of a specific “immune signature” (i.e., high or low concentrations of one or more specific chemokine) of COVID-19 patients would potentially represent a helpful clinical tool for early identification, already in the early stages of the infection, of patients more or less prone to develop a severe clinical condition (11).

Evidences accumulated through the last year, showed that a complex interplay involving several components of the immune system, is mandatory for the onset and progression of the CS. These components include cytokines, chemokines and immune-active infiltrating cells. It is important highlighting that the three components exert different but ultimately convergent roles each of which is mandatory for the onset of CS. In other words, the contribution of specific cytokines, chemokines, and immune-active infiltrating cells could be regarded as a rate limiting step of the process, in that the lack of interplay between any of these components would prevent the CS (10).

Based on the above notions and taking into account the currently available data, a cardinal prototype for each component appears to have been identified. At present, IL-6, CXCL10, and infiltrating macrophages are regarded as the principal player for cytokines, chemokines, and immune-active infiltrating cells, respectively.

The present review will be aimed at providing an overview of the mechanisms by which the three above described components interact with each other and of the specific role of IL-6, CXCL10, and macrophages in the pathogenesis of CS.

## Cytokines and Chemokines: Differences and Similarities

Cytokines and chemokines, due to the common knowledge that chemokines are indeed a family of cytokines, may in some cases erroneously considered to be and act as “the same thing.” However, cytokines and chemokines do differ for at least some aspects, indeed they both play crucial but different roles during the inflammatory process. Before addressing the main difference between cytokines and chemokines a brief description of these two group of molecules should be provided. Cytokines are a broad and loose category of low molecular weight proteins (~5–20 kDa), mainly involved in cell signaling. They are secreted by different types of cells, affecting the behavior of other cells, often including the releasing cells themselves. Some cytokines are able to enhance or inhibit the action of other cytokines through complex ways. Cytokines include some chemokines, interferons, interleukins, lymphokines, tumor necrosis factor (12, 13).

Chemokines are a family of small cytokines, or signaling proteins secreted by cells. Their name is derived from their ability to induce directed chemotaxis in nearby responsive cells, indeed they are chemotactic cytokines (14, 15). The chemotactic process promoted by chemokines is due to the binding of chemokines to specific 7 transmembrane G protein receptors expressed on target cells (12, 15–18). Chemokines can be chemically identified by their small size and by a four cysteine residues in conserved locations that are key to forming their 3-dimensional shape. Chemokines have been classified into four main subfamilies: “CXC, CC, CX3C, and XC” (12, 14).

In order to understand the complex interplay between cytokines and chemokines, it should be remembered that any inflammatory process is characterized by the presence of both cytokines and chemokines.

Indeed, in the early phase of an acute inflammatory event, as for example lung inflammation, the migration of leukocytes, neutrophils and other immune cells is one of the first events from which lung inflammation will further propagate (19). These immune cells (which in the specific case of lung are mainly neutrophils) undergo directed migration along “chemotactic” gradients to the inflamed site (19). This chemotactic gradient is orchestrated by chemokines secreted by endothelial cells, resident stromal cells, and parenchyma cells. The chemokine milieu largely determines both the type (macrophages, leukocytes, neutrophils) of cell infiltrate and the amount of infiltrating cells which will be recruited to the site of inflammation (20). This may happen thanks to the chemotactic action mediated by the binding of chemokines with their specific chemokines-receptors expressed on the immune-cell surface. The chemokine/chemokine receptor system is a highly redundant one in that, one chemokines binds to more than one receptor and one receptor interacts with multiple chemokines. Some exceptions to this general rule exist, such as the exclusive interaction between the CXCR3 receptor and its ligands (CXCL9, CXCL10, CXCL11) (14). More recently, the general concept of redundancy was re-discussed as several evidences pointed against redundancy of actual biological function in the chemokine system (21). The cell recruitment by chemokines/chemokines receptor binding, leads to the activation of immune cells which, in turn, will release several cytokines (19, 20, 22). The subsequent events of cellular/cytokine interactions are crucial for initiating and propagating the inflammatory response that leads to pulmonary injury (19). Both TNFα and IL-1 are early-response cytokines that are necessary not only for the initiation of acute inflammation, but are also required for perpetuation of the inflammatory response, leading to a chronic inflammatory state (23, 24). This event is paralleled by the production, by the major cellular components of the alveolar-capillary membrane or airway of the lung, of chemokines which actively contribute to the inflammatory response, being critical for the orchestration of the directed migration of leukocytes into the lung (19, 25). The fact that, the expression of CXC chemokines by the cellular constituents of the lung is stimulus specific should be highlighted. In particular, during SARS-COV-2 infection, the active replication and release of the *virus* cause the host cell to undergo pyro-ptosis and release several damage- associated-molecular-patterns (i.e., ATP, oligomers and nucleic acids) which “stimulate” epithelial cells, endothelial cells and alveolar macrophages, to secrete pro-inflammatory cytokines and chemokines (26–28). Chemokines attract monocytes, macrophages and T cells to the site of infection. These cells further promote the progression of inflammation by releasing IFN-γ as well as other pro- inflammatory cytokines establishing a pro-inflammatory feedback loop, which will result in a further production of chemokines which will recruit more inflammatory cells (27). This inflammatory loop will result in a damage of the lung architecture. In addition, the resulting cytokine storm characterized by high circulating concentrations of immune active molecules will subsequently spread to other organs, leading to multi-organ damage (10).

In the subsequent section, a description of the network occurring between IL-6, CXCL10 and macrophages in initiation and maintenance of the CS will be overviewed.

## The IL-6-CXCL10-Macrophages Network

Interleukin-6 (IL-6) is a four-helical cytokine of 184 amino acids (29) primarily produced during acute and chronic inflammation. IL-6 induces a transcriptional inflammatory response through its binding to interleukin 6 receptor, alpha (IL-6Rα) (30). IL-6 is involved in the promotion of the specific differentiation of CD4 naïve T-cells in the acquisition of the immune response, it acts on B-cells, T-cells, hepatocytes, hematopoietic progenitor cells and cells of the central nervous system. IL-6 is also required for the generation of Th17 cells (31).

IL-6 is secreted by several cells of the immune system but is mainly produced by macrophage and T cells activated by a viral or bacterial infection or by other immune cells (30, 32, 33) representing a signal for the induction of a response to the infection by cells of the immune system (30, 34).

Besides being produced by macrophages (35), IL-6 is also produced by a variety of different resident cells including keratinocytes, enterocytes, hepatocytes (33), pneumocytes, and bronchial epithelial cell (36), smooth muscle cells (37), skeletal muscle cells (38), osteoblasts (39), adipocytes (40), neurons (35, 41). Interestingly IL-6 was also shown to be produced by lung epithelial cells in response to a variety of different stimuli including allergens, respiratory virus and exercise (42–44). A number of studies have shown an overexpression of IL-6 in bronchial epithelial cells in patients (adult and children) with asthma (42–45).

As far as COVID-19 is concerned, evidences have accumulated supporting the concept that IL-6 plays a major role in the cytokine storm. The so-called COVID-19–related cytokine storm is a potentially fatal immune reaction induced by hyper-production -activation of T cells, during which a strong induction of IL-6 secretion is observed (46, 47). The consequent high levels of IL-6 (together with other factors), act on endothelial cells of lung capillaries, by increasing their permeability for serum proteins and improving the transmigration of inflammatory cells from vessels, leading, in more severe cases of COVID-19, to an uncontrolled excessive immune-response (46, 47).

In the attempt to identify a possible pharmacological strategy against SARS-COV-2, a clinical trial tested the ability of tocilizumab, a monoclonal antibody to inhibit the biological effects of IL-6 (48). However, more recent clinical data did not support the use of Tocilizumab in these patients since it caused several adverse effects including neutropenia, transaminitis and immunosuppression, increased risk of secondary infection, liver dysfunction, and cytopenias (3).

Nevertheless, apart from tocilizumab, other several monoclonal antibodies potentially preventing the biological effects of IL-6 (like Sarilumab, Siltuximab, Sirukumab, Clazakizumabo, Olokizumab, Levilimab) are under investigation or being tested in clinical trials (49). More interestingly, also a broad spectrum of cytokines/chemokines inhibitors not specifically targeting IL-6 and its secretion pathways was applied to counteract COVID-19. Just to give few examples Baricitinib (a JAKs inhibitor), was found to reduce the development of cytokine storm in animals and patients with COVID-19 (50). Another JAK1/JAK2 inhibitor, Ruxolitinib, was shown to ameliorate pulmonary function in COVID-19 patients (51). Other compounds (i.e., steroids, Desametasone) inducing the inhibition of the NF-kB pathway, predominantly reduced highly pro-inflammatory cytokines and chemokines, involved in aberrant systemic inflammatory responses of COVID-19 (52). Baricitinib (JAKs inhibitor) in combination with Remdesivir (NFKb inhibitor) resulted in reduced hospitalization period and accelerated recovery time in critically ill patients compared to Remdesivir alone 33306283. These data would suggest that combination therapy with JAK inhibitors and other agents with the potential to normalize NFkB- signaling, such as Ruxolitinib, Remdesivir or TNF antagonists, may be a better therapeutic approach than monotherapy alone (53). Therefore, although several clinical and experimental evidences, accumulated through the last few months, consistently identified IL-6 as a crucial molecule involved in the Sars-Cov-2-related CS representing a cardinal mediator of the adverse clinical consequences, it should be highlighted that a number of other factors are required in order to orchestrate all the events that take place in the onset and progression of the CS. Indeed, the interplay between IL-6, its cellular source and specific chemokines recruiting these latter are all mandatory and limiting steps required for the initiation and perpetuation of the CS of COVID-19. As a matter of fact, consolidated data from literature showed that IL-6 is secreted by lung resident cells after a stimulus (i.e., viral or other infection) but this is likely just one step of a much more complex scale of events that deserves to be elucidated in order to understand what actually happens in the early phase of COVID-19. Following SARS-COV-2 infection, both lung resident cells and cells of the immune response contribute to increasing the levels of IL-6 in lungs and consequently in serum of affected patients.

In addition to other well-known biological effects, IL-6 signal transduction induced by binding to immune cells expressing its receptor (α-IL-6R) activates the JAK/STAT kinase pathway leading to further production of several cytokines and chemokines (54). These latter include, CCL2 and CXCL8 causing also an increase in the expression of small protein such as E−cadherin and VEGF (vascular endothelial growth factor) (55–59).

The above molecules do play a specific and relevant role in the onset of CS. Indeed, on one hand VEGF and E−cadherin by increasing vascular permeability and leakage, will facilitate immune cells migration and trafficking from vessels to lung, ultimately favoring lung dysfunction and respiratory disease (55, 56). On the other hand, this event is paralleled by an increase of specific chemokines which will further recruit immune cells. CCL2 is a chemoattractant for monocytes, dendritic cells, and memory T cells expressing its receptor CCR2 (60), while CXCL8, is secreted by monocytes/macrophage, serving as a powerful chemoattractant for neutrophils expressing its receptors CXCR1 and CXCR2 (61). The secretion of these chemokines fits with the notion that a predominant presence of peripherally derived monocytes, neutrophils, and macrophages was found in BALF of severe cases of COVID-19 and in post mortem autopsies (62). Besides neutrophils and monocytes (mainly attracted by CCL2 and CXCL8), macrophages represent a predominant cell type characterizing lung infiltrate of COVID-19 patients, being a direct target of SARS-COV-2 owing to their abundant expression of ACE-2 and TMPRSS2 (63). Thus, also macrophages seem to play a major role in the disease, also in view of the evidence that macrophages represent one of the main source of IL-6 secretion (29, 30, 35, 64).

Thus, the predominance of infiltrating macrophages would close the link between IL-6 (macrophages secretory product) and CXCL10 (powerful recruiter of macrophages). CXCL10 was recently identified as the cardinal chemokine playing a crucial role in COVID-19 being a chemoattractant for monocytes/macrophages, dendritic cells, NK cells, and T cells. Elevated serum levels of CXCL10 were consistently reported in patients with COVID-19, being positively correlated (together with CCL2) with increased disease severity and, more importantly with an increased risk of mortality (11, 65, 66). High levels of CXCL10 were previously reported to be associated also with the severe acute respiratory syndrome (SARS) disease progression and to the development of ARDS in preclinical models (67, 68).

**Figure 1:**
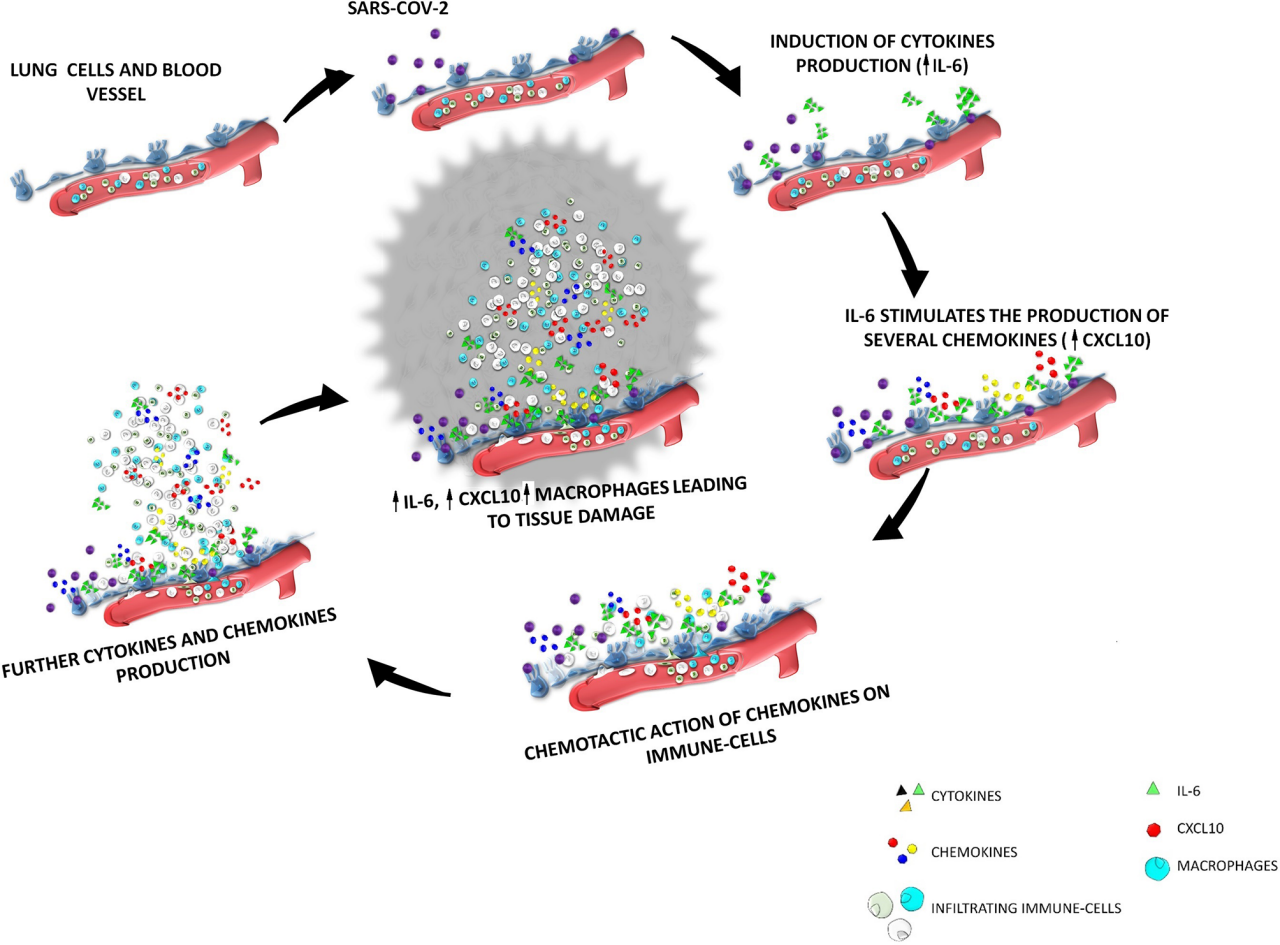
The cytokine storm after SARS-COV-2 infection the interplay among IL-6-CXCL10-macrophages. SARS-COV-2 enters the respiratory tract and binds to ACE-2 receptors expressed by lung epithelial cells. After virus entrance and binding, a sequence of events will start inside the lung interstitium; SARS-COV-2 induce in the lung epithelium the production of cytokines among which IL-6 is the mainly secreted; IL-6 stimulates the production of several chemokines (including CXCL10); the increase of the secretion of these chemokines will induce a chemotactic action on immune-cells of the blood circulation, which will be recruited from vessels to interstitium; the increase of immune cells in the lung interstitium lead to an increase of the production of further cytokine and chemokines; in particular an increased production of CXCL10 induced by IL-6 lead to an increase infiltration of macrophages which are the main source of IL-6, thus generating a loop vicious circle characterized by an hyper-production of IL-6 by lung resident cells as well as by macrophages recruited by CXCL10.

Thus, increased circulating concentrations of CXCL10 seem to characterize both SARS-COV-1 and SARS-COV-2 infections. In *ex vivo* human lung tissue explants, the inoculation of SASR-COV-1 and SARS-COV-2 up-regulated the expression of different chemokines/cytokines, being the secretion of CXCL10 strongly enhanced in both cases (69). Furthermore, CXCL10 circulating levels were correlated with disease severity in both SARS-COV-1 and SARS-COV-2 infections (10, 69). It seems worth highlighting that, according to previous studies, patients with severe SARS-COV-1 were found to display a CXCL10 mean level of 10000 pg/ml (70) versus 5000 pg/ml in patients with severe SARS-2 (71). In conclusion, the recruitment of high levels of CXCR3-expressing macrophages by CXCL10, would lead to the production of further IL-6 in the lungs. Thus, the initial cytokine/chemokine response would be perpetuated leading to a kind of loop vicious circle characterized by an hyper-production of IL-6 by lung resident cells as well as by infiltrating immune cells (mainly macrophages) recruited by specific chemokines (mainly CXCL10). This sequence of events will perpetuate the recruitment of immune-active cells. A schematic representation of the above scenario is shown in **Figure 1**. This fulsome immune response represents the *primum movens* of the cytokine storm, which, in more severe cases, promotes the damage of resident cells and subsequent organ failure.

## Conclusions

The common effort made by scientists in the last few months, provided more insights into the mechanisms involved in the “cytokine storm,” a peculiar and worrisome event of SARS-COV-2 infection. According to currently available studies, a number of cytokines (such as IL-6, TNFα, IFNγ, and others) chemokines (such as CCL2, CXCL8, CXCL10 and others) and infiltrating cells (such as neutrophils, monocytes, t-cells, macrophages and others) were shown to be involved in the immune mechanisms sustaining CS. A summary of the studies addressing the role of the here overviewed chemokines and cytokines is provided in **Table 1**. Several of the above listed factors were found to be correlated with a more or less severe course of the disease as well as to the patient’s overall risk.

**Table 1:** Summary of studies showing a role for the here overviewed cytokines and chemokines in COVID-19.

Cytokines and Chemokines	Main studies	Reference number
**IL-6**	Tanaka et al., Khadke et al., Masià et al., Patel et al.; Choudhary et al., Chen et al.	(3, 47–49, 54, 56)
**CCL2**	Chua et al; Liao et al., Yang et al, Blanco-melo et al.	(52, 62, 65, 66)
**CXCL8**	Chen et al.; Chua et al; Liao et al., Blanco melo et al.	(26, 52, 62, 66)
**CXCL10**	Yang et al., Blanco Melo et al, Chu et al., Blot et al	(65, 66, 69, 71)

It is now clear that the action of a single component of the immune system (a given cytokine, chemokine, or immune cell type) would not be sufficient to fully drive the adverse events characterizing the CS, but it is rather the interplay of these distinct components that is crucial for driving the final detrimental effect. Indeed, the concomitant presence of cytokines, chemokines, and infiltrating immune cells is a mandatory condition for the development of the sequence of events leading to CS and the related clinical consequences. Based on current knowledge, it would seem that more than one cytokine, chemokine, or infiltrating cell type is involved; however, at present, the interplay among IL-6, CXCL10, and macrophages, could represent a main circuit for the onset maintenance and progression of CS in COVID-19. However further *in vitro* and *in vivo* studies are needed to better clarify this issue and to confirm the here proposed main role of these three components. As a last consideration, it seems worth highlighting that CS is likely not the sole responsible for a severe course of COVID-19. Indeed, since the beginning of the pandemic, a wide spectrum of other factors including age, gender and, more importantly the presence of comorbidities, and likely others yet to be identified, all contribute to the outcome of Sars-CoV-2 infection. The present review was not aimed at fully elucidating the complex mechanisms sustaining the cytokine storm in COVID-19, but rather at providing more insights into the currently identified “prime actors” involved in this scenario. The here overviewed evidences might provide a food for thought for future studies aimed at further clarifying the relative role of each component of the immune response to SARS-COV-2 infection, as well as to develop potential therapeutic strategies.

## Author Contributions

All authors listed have made a substantial, direct, and intellectual contribution to the work and approved it for publication.

## Conflict of Interest

The authors declare that the research was conducted in the absence of any commercial or financial relationships that could be construed as a potential conflict of interest.

## References

[1] Turner AJ Tipnis SR Guy JL Rice G Hooper NM. ACEH/ACE2 is a Novel Mammalian Metallocarboxypeptidase and a Homologue of Angiotensin-Converting Enzyme Insensitive to ACE Inhibitors. Can J Physiol Pharmacol (2002) 80(4):346–53. 10.1139/y02-021 12025971

[2] Rotondi M Coperchini F Ricci G Denegri M Croce L Ngnitejeu ST. Detection of SARS-COV-2 Receptor ACE-2 mRNA in Thyroid Cells: A Clue for COVID-19-related Subacute Thyroiditis. J Endocrinol Invest (2020) 44(5):1085–90. 10.1007/s40618-020-01436-w PMC7538193 33025553

[3] Khadke S Ahmed N Ratts R Raju S Gallogly M de Lima M. Harnessing the Immune System to Overcome Cytokine Storm and Reduce Viral Load in COVID-19: A Review of the Phases of Illness and Therapeutic Agents. Virol J (2020) 17(1):154. 10.1186/s12985-020-01415-w 33059711 PMC7558250

[4] Devaux CA Rolain JM Raoult D. ACE2 Receptor Polymorphism: Susceptibility to SARS-CoV-2, Hypertension, Multi-Organ Failure, and COVID-19 Disease Outcome. J Microbiol Immunol Infect (2020) 53(3):425–35. 10.1016/j.jmii.2020.04.015 PMC7201239 32414646

[5] Hamming I Timens W Bulthuis ML Lely AT Navis G van Goor H. Tissue Distribution of ACE2 Protein, the Functional Receptor for SARS Coronavirus. A First Step in Understanding SARS Pathogenesis. J Pathol (2004) 203(2):631–7. 10.1002/path.1570 PMC7167720 15141377

[6] Hoffmann M Kleine-Weber H Schroeder S Krüger N Herrler T Erichsen S. SARS-Cov-2 Cell Entry Depends on ACE2 and TMPRSS2 and Is Blocked by a Clinically Proven Protease Inhibitor. Cell (2020) 181(2):271–80. 10.1016/j.cell.2020.02.052 PMC7102627 32142651

[7] Li MY Li L Zhang Y Wang XS. Expression of the SARS-CoV-2 Cell Receptor Gene ACE2 in a Wide Variety of Human Tissues. Infect Dis Poverty (2020) 9(1):45. 10.1186/s40249-020-00662-x 32345362 PMC7186534

[8] Cecconi M Forni G Mantovani A. Ten Things We Learned About COVID-19. Intensive Care Med (2020) 46(8):1590–3. 10.1007/s00134-020-06140-0 PMC7273118 32504103

[9] Oliviero A de Castro F Coperchini F Chiovato L Rotondi M. Covid-19 Pulmonary and Olfactory Dysfunctions: Is the Chemokine CXCL10 the Common Denominator? Neuroscientist (2020), 1073858420939033. 10.1177/1073858420939033 32659199

[10] Coperchini F Chiovato L Croce L Magri F Rotondi M. The Cytokine Storm in COVID-19: An Overview of the Involvement of the Chemokine/Chemokine-Receptor System. Cytokine Growth Factor Rev (2020) 53:25–32. 10.1016/j.cytogfr.2020.05.003 32446778 PMC7211650

[11] Coperchini F Chiovato L Ricci G Croce L Magri F Rotondi M. The Cytokine Storm in COVID-19: Further Advances in Our Understanding the Role of Specific Chemokines Involved. Cytokine Growth Factor Rev (2021) 58:82–91. 10.1016/j.cytogfr.2020.12.005 33573850 PMC7837329

[12] Turner MD Nedjai B Hurst T Pennington DJ. Cytokines and Chemokines: At the Crossroads of Cell Signalling and Inflammatory Disease. Biochim Biophys Acta (2014) 1843(11):2563–82. 10.1016/j.bbamcr.2014.05.014 24892271

[13] Arend WP Palmer G Gabay C. Il-1, IL-18, and IL-33 Families of Cytokines. Immunol Rev (2008) 223:20–38. 10.1111/j.1600-065X.2008.00624.x 18613828

[14] Rotondi M Chiovato L Romagnani S Serio M Romagnani P. Role of Chemokines in Endocrine Autoimmune Diseases. Endocr Rev (2007) 28(5):492–520. 10.1210/er.2006-0044 17475924

[15] Coperchini F Croce L Marinò M Chiovato L Rotondi M. Role of Chemokine Receptors in Thyroid Cancer and Immunotherapy. Endocr Relat Cancer (2019) 26(8):R465–78. 10.1530/ERC-19-0163 31146261

[16] Bacon K Baggiolini M Broxmeyer H Horuk R Lindley I Mantovani A. Chemokine/chemokine receptor nomenclature. J Interferon Cytokine Res (2002) 22(10):1067–8. 10.1089/107999002760624305 12433287

[17] Mantovani A Savino B Locati M Zammataro L Allavena P Bonecchi R. The Chemokine System in Cancer Biology and Therapy. Cytokine Growth Factor Rev (2010) 21(1):27–39. 10.1016/j.cytogfr.2009.11.007 20004131

[18] Sozzani S Allavena P Vecchi A Mantovani A. The Role of Chemokines in the Regulation of Dendritic Cell Trafficking. J Leukoc Biol (1999) 66(1):1–9. 10.1002/jlb.66.1.1 10410984

[19] Strieter RM Kunkel SL Keane MP Standiford TJ. Chemokines in Lung Injury: Thomas a. Neff Lecture Chest (1999) 116(1 Suppl):103S–10S. 10.1378/chest.116.suppl_1.103S 10424625

[20] Luster AD. Chemokines–Chemotactic Cytokines That Mediate Inflammation. N Engl J Med (1998) 338(7):436–45. 10.1056/NEJM199802123380706 9459648

[21] Rotondi M Chiovato L. The Chemokine System as a Therapeutic Target in Autoimmune Thyroid Diseases: A Focus on the Interferon-γ Inducible Chemokines and Their Receptor. Curr Pharm Des (2011) 17(29):3202–16. 10.2174/138161211798157559 21864266

[22] Rollins BJ. Chemokines. Blood (1997) 90(3):909–28. 10.1182/blood.V90.3.909 9242519

[23] Eisenberg SP Brewer MT Verderber E Heimdal P Brandhuber BJ Thompson RC. Interleukin 1 Receptor Antagonist is a Member of the Interleukin 1 Gene Family: Evolution of a Cytokine Control Mechanism. Proc Natl Acad Sci USA (1991) 88(12):5232–6. 10.1073/pnas.88.12.5232 PMC51846 1828896

[24] Le J Vilcek J. Tumor Necrosis Factor and Interleukin 1: Cytokines With Multiple Overlapping Biological Activities. Lab Invest (1987) 56(3):234–48. 3029503

[25] Strieter RM Kunkel SL Standiford T. Chemokines in the Lung; Lenfant, C., Ed.; Lung Biology in Health and Disease. Boca Raton, FL, USA: CRC Press (2003) p. 1–341. [Google Scholar]. 10.1201/b14091

[26] Chen N Zhou M Dong X Qu J Gong F Han Y. Epidemiological and Clinical Characteristics of 99 Cases of 2019 Novel Coronavirus Pneumonia in Wuhan, China: A Descriptive Study. Lancet (2020) 395:507–5139. 10.1016/S0140-6736(20)30211-7 32007143 PMC7135076

[27] Tay MZ Poh CM Rénia L MacAry PA Ng LFP. The Trinity of COVID-19: Immunity, Inflammation and Intervention. Nat Rev Immunol (2020) 20(6):363–74. 10.1038/s41577-020-0311-8 PMC7187672 32346093

[28] Huang C Wang Y Li X Ren L Zhao J Hu Y. Clinical Features of Patients Infected With 2019 Novel Coronavirus in Wuhan, China. Lancet (2020) 395(10223):497–506. 10.1016/S0140-6736(20)30183-5 31986264 PMC7159299

[29] Kishimoto T. Il-6: From its Discovery to Clinical Applications. Int Immunol (2010) 22(5):347–52. 10.1093/intimm/dxq030 20410258

[30] Kishimoto T. Interleukin-6: From Basic Science to Medicine–40 Years in Immunology. Annu Rev Immunol (2005) 23:1–21. 10.1146/annurev.immunol.23.021704.115806 15771564

[31] Kimura A Kishimoto T. Il-6: Regulator of Treg/Th17 Balance. Eur J Immunol (2010) 40(7):1830–5. 10.1002/eji.201040391 20583029

[32] Calabrese LH Rose-John S. IL-6 Biology: Implications for Clinical Targeting in Rheumatic Disease. Nat Rev Rheumatol (2014) 10(12):720–7. 10.1038/nrrheum.2014.127 25136784

[33] Schmidt-Arras D Rose-John S. IL-6 Pathway in the Liver: From Physiopathology to Therapy. J Hepatol (2016) 64(6):1403–15. 10.1016/j.jhep.2016.02.004 26867490

[34] Schaper F Rose-John S. Interleukin-6: Biology, Signaling and Strategies of Blockade. Cytokine Growth Factor Rev (2015) 26(5):475–87. 10.1016/j.cytogfr.2015.07.004 26189695

[35] Shapouri-Moghaddam A Mohammadian S Vazini H Taghadosi M Esmaeili SA Mardani F. Macrophage Plasticity, Polarization, and Function in Health and Disease. J Cell Physiol (2018) 233(9):6425–40. 10.1002/jcp.26429 PMC13482560 29319160

[36] Cheung CY Poon LL Ng IH Luk W Sia SF Wu MH. Cytokine Responses in Severe Acute Respiratory Syndrome Coronavirus-Infected Macrophages In Vitro: Possible Relevance to Pathogenesis. J Virol (2005) 79(12):7819–26. 10.1128/JVI.79.12.7819-7826.2005 PMC1143636 15919935

[37] Kyotani Y Takasawa S Yoshizumi M. Proliferative Pathways of Vascular Smooth Muscle Cells in Response to Intermittent Hypoxia. Int J Mol Sci (2019) 20(11):2706. 10.3390/ijms20112706 PMC6600262 31159449

[38] Barbalho SM Prado Neto EV De Alvares Goulart R Bechara MD Baisi Chagas EF Audi M. Myokines: A Descriptive Review. J Sports Med Phys Fitness (2020) 60(12):1583–90. 10.23736/S0022-4707.20.10884-3 32586076

[39] Kovács B Vajda E Nagy EE. Regulatory Effects and Interactions of the Wnt and OPG-RANKL-RANK Signaling At the Bone-Cartilage Interface in Osteoarthritis. Int J Mol Sci (2019) 20(18):4653. 10.3390/ijms20184653 PMC6769977 31546898

[40] Xie C Chen Q. Adipokines: New Therapeutic Target for Osteoarthritis? Curr Rheumatol Rep (2019) 21(12):71. 10.1007/s11926-019-0868-z 31813080 PMC7291783

[41] Brábek J Jakubek M Vellieux F Novotný J Kolář M Lacina L. Interleukin-6: Molecule in the Intersection of Cancer, Ageing and COVID-19. Int J Mol Sci (2020) 21(21):7937. 10.3390/ijms21217937 PMC7662856 33114676

[42] Marini M Vittori E Hollemborg J Mattoli S. Expression of the Potent Inflammatory Cytokines, Granulocyte-Macrophage-Colony-Stimulating Factor and Interleukin-6 and interleukin-8, in Bronchial Epithelial Cells of Patients With Asthma. J Allergy Clin Immunol (1992) 89(5):1001–9. 10.1016/0091-6749(92)90223-O 1583242

[43] Yokoyama A Kohno N Fujino S Hamada H Inoue Y Fujioka S. Circulating Interleukin-6 Levels in Patients With Bronchial Asthma. Am J Respir Crit Care Med (1995) 151(5):1354–8. 10.1164/ajrccm.151.5.7735584 7735584

[44] Broide DH Lotz M Cuomo AJ Coburn DA Federman EC Wasserman SI. Cytokines in Symptomatic Asthma Airways. J Allergy Clin Immunol (1992) 89(5):958–67. 10.1016/0091-6749(92)90218-Q 1374772

[45] Rincon M Irvin CG. Role of IL-6 in Asthma and Other Inflammatory Pulmonary Diseases. Int J Biol Sci (2012) 8(9):1281–90. 10.7150/ijbs.4874 PMC3491451 23136556

[46] Tanaka T Narazaki M Kishimoto T. IL-6 in Inflammation, Immunity, and Disease. Cold Spring Harb Perspect Biol (2014) 6(10):a016295. 10.1101/cshperspect.a016295 25190079 PMC4176007

[47] Tanaka T Narazaki M Kishimoto T. Immunotherapeutic Implications of IL-6 Blockade for Cytokine Storm. Immunotherapy (2016) 8(8):959–70. 10.2217/imt-2016-0020 27381687

[48] Masiá M Fernández-González M Padilla S Ortega P García JA Agulló V. Impact of Interleukin-6 Blockade With Tocilizumab on SARS-CoV-2 Viral Kinetics and Antibody Responses in Patients With COVID-19: A Prospective Cohort Study. EBioMedicine (2020) 60:102999. 10.1016/j.ebiom.2020.102999 32950003 PMC7492814

[49] Patel S Saxena B Mehta P. Recent Updates in the Clinical Trials of Therapeutic Monoclonal Antibodies Targeting Cytokine Storm for the Management of COVID-19. Heliyon (2021) 7(2):e06158. 10.1016/j.heliyon.2021.e06158 33553708 PMC7846241

[50] Hoang TN Pino M Boddapati AK Viox EG Starke CE Upadhyay AA. Baricitinib Treatment Resolves Lower-Airway Macrophage Inflammation and Neutrophil Recruitment in SARS-CoV-2-infected Rhesus Macaques. Cell (2021) 184(2):460–75. 10.1016/j.cell.2020.11.007 PMC7654323 33278358

[51] Vannucchi AM Sordi B Morettini A Nozzoli C Poggesi L Pieralli F. Compassionate Use of JAK1/2 Inhibitor Ruxolitinib for Severe COVID-19: A Prospective Observational Study. Leukemia (2020) 35(4):1121–33. 10.1038/s41375-020-01018-y PMC7437386 32814839

[52] Chua RL Lukassen S Trump S Hennig BP Wendisch D Pott F. Covid-19 Severity Correlates With Airway Epithelium-Immune Cell Interactions Identified by Single-Cell Analysis. Nat Biotechnol (2020) 38(8):970–9. 10.1038/s41587-020-0602-4 32591762

[53] Yan B Freiwald T Chauss D Wang L West E Bibby J. Sars-CoV2 Drives JAK1/2-dependent Local and Systemic Complement Hyper-Activation. Res Sq (2020). 10.21203/rs.3.rs-33390/v1

[54] Choudhary S Sharma K Silakari O. The Interplay Between Inflammatory Pathways and COVID-19: A Critical Review on Pathogenesis and Therapeutic Options. Microb Pathog (2021) 150:104673. 10.1016/j.micpath.2020.104673 33278517 PMC7709793

[55] Johnson DE O’Keefe RA Grandis JR. Targeting the IL-6/JAK/STAT3 Signalling Axis in Cancer. Nat Rev Clin Oncol (2018) 15(4):234–48. 10.1038/nrclinonc.2018.8 PMC5858971 29405201

[56] Chen JJ Zhang LN Hou H Xu L Ji K. Interleukin-6 Signaling Blockade Treatment for Cytokine Release Syndrome in COVID-19 (Review). Exp Ther Med (2021) 21(1):24. 10.3892/etm.2020.9456 33262810 PMC7690237

[57] Hurst SM Wilkinson TS McLoughlin RM Jones S Horiuchi S Yamamoto N. Il-6 and its Soluble Receptor Orchestrate a Temporal Switch in the Pattern of Leukocyte Recruitment Seen During Acute Inflammation. Immunity (2001) 14(6):705–14. 10.1016/S1074-7613(01)00151-0 11420041

[58] Marin V Montero-Julian FA Grès S Boulay V Bongrand P Farnarier C. The IL-6-soluble Il-6Ralpha Autocrine Loop of Endothelial Activation as an Intermediate Between Acute and Chronic Inflammation: An Experimental Model Involving Thrombin. J Immunol (2001) 167(6):3435–42. 10.4049/jimmunol.167.6.3435 11544336

[59] Gabay C. Interleukin-6 and Chronic Inflammation. Arthritis Res Ther (2006) 8(Suppl 2):S3. 10.1186/ar1917 PMC3226076 16899107

[60] Van Coillie E Van Damme J Opdenakker G. The MCP/eotaxin Subfamily of CC Chemokines. Cytokine Growth Factor Rev (1999) 10(1):61–86. 10.1016/S1359-6101(99)00005-2 10379912

[61] Ha H Debnath B Neamati N. Role of the CXCL8-CXCR1/2 Axis in Cancer and Inflammatory Diseases. Theranostics (2017) 7(6):1543–88. 10.7150/thno.15625 PMC5436513 28529637

[62] Liao M Liu Y Yuan J Wen Y Xu G Zhao J. Single-Cell Landscape of Bronchoalveolar Immune Cells in Patients With COVID-19. Nat Med (2020) 26(6):842–4. 10.1038/s41591-020-0901-9 32398875

[63] Carsana L Sonzogni A Nasr A Rossi RS Pellegrinelli A Zerbi P. Pulmonary Post-Mortem Findings in a Series of COVID-19 Cases From Northern Italy: A Two-Centre Descriptive Study. Lancet Infect Dis (2020) 20(10):1135–40. 10.1016/S1473-3099(20)30434-5 PMC7279758 32526193

[64] Hirano T. Interleukin 6 and its Receptor: Ten Years Later. Int Rev Immunol (1998) 16(3-4):249–84. 10.3109/08830189809042997 9505191

[65] Yang L Han Y Nilsson-Payant BE Gupta V Wang P Duan X. A Human Pluripotent Stem Cell-Based Platform to Study SARS-Cov-2 Tropism and Model Virus Infection in Human Cells and Organoids. Cell Stem Cell (2020) 27(1):125–36. 10.1016/j.stem.2020.06.015 PMC7303620 32579880

[66] Blanco-Melo D Nilsson-Payant BE Liu WC Uhl S Hoagland D Møller R. Imbalanced Host Response to SARS-CoV-2 Drives Development of COVID-19. Cell (2020) 181(5):1036–45. 10.1016/j.cell.2020.04.026 PMC7227586 32416070

[67] Altara R Manca M Brandão RD Zeidan A Booz GW Zouein FA. Emerging Importance of Chemokine Receptor CXCR3 and its Ligands in Cardiovascular Diseases. Clin Sci (Lond) (2016) 130(7):463–78. 10.1042/CS20150666 26888559

[68] Jiang Y Xu J Zhou C Wu Z Zhong S Liu J. Characterization of Cytokine/Chemokine Profiles of Severe Acute Respiratory Syndrome. Am J Respir Crit Care Med (2005) 171(8):850–7. 10.1164/rccm.200407-857OC 15657466

[69] Chu H Chan JF Wang Y Yuen TT Chai Y Hou Y. Comparative Replication and Immune Activation Profiles of SARS-CoV-2 and SARS-CoV in Human Lungs: An Ex Vivo Study With Implications for the Pathogenesis of COVID-19. Clin Infect Dis (2020) 71(6):1400–9. 10.1093/cid/ciaa410 PMC7184390 32270184

[70] Wong CK Lam CW Wu AK Ip WK Lee NL Chan IH. Plasma Inflammatory Cytokines and Chemokines in Severe Acute Respiratory Syndrome. Clin Exp Immunol (2004) 136(1):95–103. 10.1111/j.1365-2249.2004.02415.x 15030519 PMC1808997

[71] Blot M Jacquier M Aho Glele LS Beltramo G Nguyen M Bonniaud P. CXCL10 Could Drive Longer Duration of Mechanical Ventilation During COVID-19 Ards. Crit Care (2020) 24(1):632. 10.1186/s13054-020-03328-0 33138839 PMC7604548

